# From Latent Manifolds to Targeted Molecular Probes: An Interpretable, Kinome-Scale Generative Machine Learning Framework for Family-Based Kinase Ligand Design

**DOI:** 10.3390/biom16020209

**Published:** 2026-01-29

**Authors:** Gennady Verkhivker, Ryan Kassab, Keerthi Krishnan

**Affiliations:** 1 Graduate Program in Computational and Data Sciences, Keck Center for Science and Engineering, Schmid College of Science and Technology, Chapman University, Orange, CA 92866, USA; rkassab@chapman.edu (R.K.); kekrishnan@chapman.edu (K.K.); 2 Department of Biomedical and Pharmaceutical Sciences, Chapman University School of Pharmacy, Irvine, CA 92618, USA

**Keywords:** autonomous molecular design, deep learning models, latent space landscapes, protein kinases, kinase ligands, local neighborhood sampling chemical modeling, kinase association likelihood classifiers, explainable machine learning

## Abstract

Scaffold-aware artificial intelligence (AI) models enable systematic exploration of chemical space conditioned on protein-interacting ligands, yet the representational principles governing their behavior remain poorly understood. The computational representation of structurally complex kinase small molecules remains a formidable challenge due to the high conservation of ATP active site architecture across the kinome and the topological complexity of structural scaffolds in current generative AI frameworks. In this study, we present a diagnostic, modular and chemistry-first generative framework for design of targeted SRC kinase ligands by integrating ChemVAE-based latent space modeling, a chemically interpretable structural similarity metric (Kinase Likelihood Score), Bayesian optimization, and cluster-guided local neighborhood sampling. Using a comprehensive dataset of protein kinase ligands, we examine scaffold topology, latent-space geometry, and model-driven generative trajectories. We show that chemically distinct scaffolds can converge toward overlapping latent representations, revealing intrinsic degeneracy in scaffold encoding, while specific topological motifs function as organizing anchors that constrain generative diversification. The results demonstrate that kinase scaffolds spanning 37 protein kinase families spontaneously organize into a coherent, low-dimensional manifold in latent space, with SRC-like scaffolds acting as a structural “hub” that enables rational scaffold transformation. Our local sampling approach successfully converts scaffolds from other kinase families (notably LCK) into novel SRC-like chemotypes, with LCK-derived molecules accounting for ~40% of high-similarity outputs. However, both generative strategies reveal a critical limitation: SMILES-based representations systematically fail to recover multi-ring aromatic systems—a topological hallmark of kinase chemotypes—despite ring count being a top feature in our structural similarity metric. This “representation gap” demonstrates that no amount of scoring refinement can compensate for a generative engine that cannot access topologically constrained regions. By diagnosing these constraints within a transparent pipeline and reframing scaffold-aware ligand design as a problem of molecular representation our work provides a conceptual framework for interpreting generative model behavior and for guiding the incorporation of structural priors into future molecular AI architectures.

## 1. Introduction

The discovery of small molecules targeting therapeutically important and structurally complex proteins such as kinases, GPCRs, has long relied on iterative cycles of chemical synthesis, high-throughput screening, and structure–activity relationship (SAR) analysis. This labor-intensive process, constrained by experimental throughput and human intuition, has often required years to yield a single clinical candidate. The past decade, however, has witnessed a profound transformation: the integration of artificial intelligence (AI) and machine learning (ML) into drug discovery has enabled de novo, property-driven generation of drug-like molecules with unprecedented speed, scale, and chemical novelty [1,2,3,4,5,6,7,8]. Many deep learning approaches have been put forward employing various neural network architectures, molecular representations, and analysis metrics for targeted compound design and their applications [9,10,11,12,13,14,15,16].

This paradigm shift evolved from syntax-aware sequence modeling toward structure- and function-aware molecular design. Early efforts treated molecules as textual sequences using the Simplified Molecular Input Line Entry System (SMILES) and applied natural language processing (NLP) techniques to chemical space. Deep neural network (DNN) models, most notably variational autoencoders (VAEs) [9] and generative adversarial networks (GANs) [17], proved particularly fruitful in molecular design [17,18,19,20,21,22,23,24,25,26,27,28]. Among the pioneering approaches were SeqGAN [18] and Objective-Reinforced GANs (ORGAN) [19], which coupled recurrent neural network (RNN) generators with discriminators trained not only to assess chemical validity but also to maximize user-defined molecular properties—a foundational step toward goal-directed generation. Subsequent advances such as LatentGAN [20], DruGAN [23], and MolGAN [29] extended these ideas to graph-structured representations, while CycleGAN-inspired methods like MolCycleGAN [30] enabled transformation rules between compound sets with differing properties [30,31]. Despite these innovations, GAN-based approaches often suffered from mode collapse and low validity, highlighting the fragility of adversarial training in discrete chemical spaces [32,33,34,35,36].

A more robust alternative emerged with ChemVAE [9], a variational autoencoder that encoded SMILES strings into a smooth, continuous 196-dimensional latent space while simultaneously predicting key drug-likeness metrics—a quantitative estimate of drug-likeness (QED) [37], synthetic accessibility score (SAS) [38], and logP [39]. This architecture enabled efficient navigation of chemical space and laid the groundwork for reward-guided generative strategies. Notably, REINVENT [20,40,41] introduced a reinforcement learning framework that fine-tuned an RNN generator toward customizable reward functions combining predicted binding affinity, QED, and scaffold similarity, thus effectively turning generative models into programmable design engines. Similarly, GENTRL used reinforcement learning over a compressed latent lattice to discover novel kinase ligands [42].

Attention-based generative models further enabled more accurate sampling from latent space and exploration of chemistry not present in training data [43]. Efficient multi-objective frameworks combined in silico property prediction with particle swarm optimization for optimal latent navigation [44,45,46], while query-based methods like QMO decoupled representation learning from guided search [47]. Despite these advances, including rigorous benchmarking of VAE, GAN, and RNN models in goal-directed and target-specific tasks [48], these approaches remained constrained by the sequential nature of SMILES, which struggles to represent cyclic and stereochemical complexity. Concurrently, transformer architectures reshaped chemical AI. Models like SMILES-BERT [49] and Chemformer [50,51] leveraged self-supervised pretraining on billions of compounds to improve generation quality and transfer learning. Alternatives such as MolDQN [52] bypassed SMILES entirely by applying deep Q-learning to graph-edit actions, yet rewards were typically computed using surrogate predictors such as random forests rather than direct protein–ligand physics, often yielding molecules that were chemically plausible but pharmacologically inert.

The limitations of 2D representations spurred interest in 3D-aware generative models [53,54]. Approaches like DeepLigBuilder [55] combined graph generative models with Monte Carlo tree search to optimize binding features, while comprehensive reviews categorized 3D generation into grid-, distance matrix-, and coordinate-based paradigms, each requiring specialized architectures [56]. However, the most transformative advances arrived with graph representation learning. Starting with Neural Message Passing (MPNN) [57], graph neural networks (GNNs) enabled direct learning from molecular topology. Chemistry-specific GNNs—such as Graph Convolutional Networks (GCNs) [58], Graph Attention Networks (GATs) [59], and Directed MPNNs (D-MPNN) [60]—demonstrated state-of-the-art performance in ADMET and bioactivity prediction, forming the backbone of interpretable frameworks like Chemprop. JT-VAE [61] further improved validity (>99%) by decomposing molecules into hierarchical junction trees, enabling precise scaffold control. Similarly, GraphAF built molecular graphs atom-by-atom with high fidelity [62], and GFlowNets introduced probabilistic sampling proportional to reward, mitigating earlier mode collapse issues [63,64].

Despite these strides, static 2D graphs remained insufficient for tasks requiring conformational awareness, such as kinase ligand design. This gap motivated the rise of geometric deep learning, where models respect rotational and translational symmetries of physical space. SchNet [65,66] pioneered continuous-filter convolutions on atomic coordinates, later refined by DimeNet++ [67,68], which incorporated directional message passing via interatomic angles. The field culminated in SE(3)-equivariant GNNs, exemplified by EquiBind [69], which predicts ligand poses by learning geometric constraints directly from structures. Complementary models like TANKBind [70] segmented proteins into functional blocks to predict interaction landscapes, while self-supervised frameworks such as GraphMVP [71] and related geometric GNNs [72,73] used contrastive learning to align 2D and 3D representations, enhancing downstream property prediction.

The current frontier integrates diffusion models and foundation architectures capable of co-designing proteins and ligands. GeoDiff [74] enabled 3D ligand generation via SE(3)-equivariant diffusion, while work by Tang et al. introduced pretrainable geometric GNNs for antibody affinity maturation [75]. Subsequent models such as TorsionDiff [76], DiffDock [77], DiffDock-L [78], and DiffLinker [79] further refined structure-aware generation and linker design. When conditioned on protein structure, diffusion-based models achieve remarkable biological specificity: RFdiffusion [80]—paired with ProteinMPNN [81]—enables de novo creation of protein binders and small-molecule scaffolds. Multimodal models like ESM3 [82] and Chroma [83] now integrate sequences, structures, and functions for zero-shot ligand generation. These advances have been accelerated by open ecosystems like PyTorch Geometric [84], DGL [85], and unified benchmarks such as the Therapeutics Data Commons (TDC) [86].

However, most modern AI tools remain black boxes, obscuring the chemical rationale behind design choices and hindering iterative refinement. Hybrid approaches, which combine the predictive power of GNNs or VAEs with chemically grounded, interpretable scoring functions, offer a promising path forward. Such frameworks enable not only generation but also diagnostic transparency, failure mode analysis, and rational scaffold transformation across kinase families. In this study, we present a diagnostic and modular generative framework for design of SRC kinase-associated ligands and targeted scaffolds. Our hybrid AI framework integrates ChemVAE-based latent space trained on ~60,000 kinase ligands spanning 37 families, with chemistry-first Kinase Association Likelihood (KAL) scorer, Bayesian optimization for global property-guided search, and cluster-guided local neighborhood sampling for scaffold-aware transformation. This work significantly extends our prior studies [87] by revealing that kinase ligands cluster on a continuous latent manifold defined by scaffold features, with SRC acting as a structural “hub” that enables rational cross-family transformation. We demonstrate that local sampling outperforms global optimization in recovering the multi-ring scaffolds essential for kinase recognition and expose a fundamental representation gap of SMILES-based generative models. Importantly, this study is not intended as a biophysical validation of binding modes, affinities, or inhibitory efficacy. Instead, we treat kinase-associated ligands as structural exemplars of scaffold families and use them to probe the representational and generative limits of scaffold-aware AI models. Here, the term “kinase-associated ligand” (or “kinase ligand”) denotes small molecules targeting kinase catalytic domains and is used in structural, chemical design and dataset-centric contexts, without necessarily implying biochemical inhibition mechanisms or pharmacological activity. Our focus is on how chemical scaffolds are encoded, transformed, and diversified in latent space, rather than on predicting or confirming functional inhibition. By combining diagnostic clarity with generative exploration, our study offers a useful interpretable ML framework for family-targeted kinase ligand design showing that scaffold-aware navigation can bridge the gap between chemical novelty and functional relevance in molecular design.

## 2. Materials and Methods

### 2.1. Data Sets of Protein Kinase Ligands and Small Molecules

To construct a robust and representative foundation for generative kinase ligand design, we assembled a large-scale, multi-source dataset that reflects the current state of kinase-targeted chemical space. Numerous large databases are available that contain molecules in a variety of representations including SMILES, 2D, and 3D. For this study, we explored the databases of generic small molecules and drug-like molecules, primarily ChEMBL [88], DrugBank [89,90], BindingDB [91], BindingMoad [92], ChEBI [93], and ZINC, a free database of commercially available compounds that contains over 230 million purchasable compounds in ready-to-dock, 3D formats [94,95,96]. Our small molecule collection integrates high confidence bioactive compounds from ChEMBL v32, DrugBank v5.1 [90], PDBbind v2023 [91] and ZINC20 [96]. To provide a meaningful contrast to kinase-biased chemistry, we sampled drug-like matter from two ultra-large enumerative databases: GDB-17 Lead-Like Set: ~11 million molecules filtered for lead-like properties (MW ≤ 450, logP ≤ 4, ≤4 HBD/HBA) [97,98], FDB-17 subset ~10 million fragment-like compounds derived from GDB-17 using synthetic accessibility and complexity filters [99]. From these, we selected ~ 220,000 diverse molecules satisfying Lipinski’s Rule of Five (MW < 700, logP ∈ [−4,6], ≤6 rotatable bonds, ≤12 HBD/HBA) and restricted to biologically relevant atoms (C, N, O, F, S, P, Cl, Br, I). This “random” background set ensures the model learns to distinguish kinase-specific pharmacophores from generic drug-like space.

For generative kinase ligand design, we assembled a comprehensive dataset of protein kinase ligands (PKIs). In 2023, Bajorath reported a total of 155,579 qualifying unique human PKIs [100]. Our curation strategy is informed by recent systematic analyses of the kinome-wide ligand landscape, including the landmark 2025 review by Koch, Kullmann, and Bajorath [101] which reports that over 206,000 protein kinase-associated small molecules have been disclosed spanning orthosteric, allosteric, and covalent mechanisms across the human kinome. For datasets of PKIs, we used ~60,000 available high-confidence PKIs. The expanded set covered the expanded set of kinase families totaling 37 distinct kinase families across the human kinome, including: SRC (SRC, LCK, FYN, YES), ABL (ABL1, ABL2), EGFR (EGFR, ERBB2/HER2, ERBB4), PDGFR (PDGFRα, PDGFRβ, KIT, CSF1R, FLT3), FGFR (FGFR1–4), INSR (INSR, IGF1R), TRK (NTRK1/2/3), ROS (ROS1, DDR1, DDR2), MET (MET, RON), RAF (ARAF, BRAF, CRAF), MLK (MAP3K9, MAP3K10, MAP3K11), LRRK (LRRK1, LRRK2), STKR (ALK, LTK, ROS, RYK), TLK (TLK1, TLK2), RIPK (RIPK1–4), WNK (WNK1–4), CLK (CLK1–4), STE20 (PAK1–7, MAP4K1–7) STE11 (MAP3K1–13), STE7 (MAP2K1–7), CAMK (CAMK1–4, DAPK1–3), DAPK, PHK (PHKG1/2), MLCK (MYLK), DCAMKL (DCAMKL1–3), MELK, BRSK, PKA (PRKACA/B/C), PKG (PRKG1/2), PKC (PRKCA–Z), AKT (AKT1–3), RSK (RPS6KA1–6), SGK (SGK1–3) CDK (CDK1–20), MAPK (MAPK1/3/8/9/11/14/p38α–δ), and GSK3 (GSK3A/B).

In the earlier study [87] we used the dataset of competitive and allosteric protein kinase ligands confirmed by X-ray crystallography that contained a total of 2899 unique ligands including 136 allosteric and 2763 orthosteric compounds with a total of 231 protein kinases [102,103,104]. In the current study, we included the latest data from the KLIFS website (accessed April 2025) that reported 4179 unique ligands confirmed by X-ray across 6738 structures for 326 kinases [105]. We also expanded the list of allosteric kinase ligands based on recent systematic analysis of X-ray structures that identified a total of 262 allosteric PK ligands [106]. For focused generative experiments on SRC, we extracted 3477 high-confidence SRC-associated ligands (IC_50_ ≤ 100 nM) and 1883 ABL1 ligands as reference scaffolds. All molecules were standardized using RDKit [107,108] with salts removed, tautomers normalized, and stereochemistry preserved. All molecules including both kinase ligands and background compounds were converted to canonical SMILES and encoded into a 196-dimensional continuous latent space using the ChemVAE architecture [9]. ChemVAE converts discrete representations of molecules to and from a multidimensional continuous representation, enabling generation of new molecules for efficient exploration and optimization via open ended chemical spaces, enabling Bayesian optimization in latent space and allowing to navigate toward regions enriched for desired properties.

### 2.2. Guided Remodeling of Latent Neighborhoods via Cluster-Directed Sampling

To enable scaffold-aware transformation of kinase ligands across families, we developed a guided latent space remodeling strategy that leverages the intrinsic structural organization of the ChemVAE embedding. Rather than applying global or random modifications, our approach performs targeted local neighborhood sampling—a process that shifts molecular representations toward chemically coherent regions of latent space while preserving scaffold integrity. We began by applying K-means clustering to the 196-dimensional ChemVAE latent space to identify functionally homogeneous neighborhoods. This unsupervised step avoids manual labeling and allows molecular embeddings to self-organize into groups based solely on structural and physicochemical similarity. We evaluated cluster configurations ranging from 2 to 5 partitions and found that a 3-cluster split yielded the highest diversity and validity of generated molecules, as well as the clearest separation of scaffold motifs (e.g., fused heterocycles vs. linear aromatics). This configuration was selected for all subsequent remodeling experiments. Within each cluster, we performed centroid-directed sampling where for every molecule with latent representation x, we computed its displacement toward the cluster centroid c using a controlled interpolation, as follows:

(1) $$\vec{x_{i}^{*}}=\vec{x_{i}}+s\left(\vec{c_{i}}-\vec{x_{i}}\right)$$

where the scaling factor governs the degree of remodeling. Given that the lower bound of s=0 corresponds to the original encoding of a given molecule, while s=1 provides us with the centroid of the cluster, this parameter was initially set to be a threshold of 0.5. By performing local sampling steps and evaluating KAL probabilities, we found that with the scaling factor s<0.5 the yield of valid molecules decreased, while a scaling factor s=0.8 remodels the molecule gradually towards the centroid of the cluster yielding valid molecules without losing information of the molecular attributes. To encourage diversity, we introduced low-magnitude isotropic noise (standard deviation = 5.0) to the remodeled vectors. Higher noise levels (≥10) degraded validity, as they pushed samples into sparse, low-decoding-density regions of the latent space. The combination of 3-cluster partitioning, centroid-directed sampling with, and minimal noise consistently produced the highest yield of valid, structurally diverse molecules. After remodeling, each vector was decoded into a SMILES string using the ChemVAE decoder. To ensure chemical plausibility, we implemented a two-stage filtering protocol: for validity screening, the decoder was run 500 times per vector; if at least one valid SMILES (as verified by RDKit) was produced, the molecule advanced. For size filtering, molecules with SMILES length < 10 were discarded to exclude trivial or non-drug-like outputs. The resulting compounds were then evaluated for KAL score, structural similarity to SRC ligands, and drug-like properties to assess the success of scaffold transformation. The GitHub site https://github.com/kassabry/Kinome-Scale-Generative-Modeling (accessed on 1 January 2026) provides detailed documentation and guides of the deposited information and software. The deep learning frameworks were supported by the TensorFlow backend [109] and python tools such as NumPy 2.4.1, scipy 1.17.0, pandas 3.0.0, and scikitlearn 1.8.0.

### 2.3. Kinase Association Likelihood Classifier

We developed the Kinase Association Likelihood (KAL), a chemistry-first, interpretable classifier that estimates the probability a molecule belongs to the chemical space of experimentally validated SRC kinase-associated ligands. The KAL score functions as an integrated chemical similarity metric rather than a predictor of biological activity. This classifier synthesizes a multidimensional feature space—including aromatic ring topology, polar surface area distribution, hydrogen-bonding patterns, and steric complementarity—into a unified assessment of structural and chemical proximity to experimentally validated SRC kinase ligands. High KAL values reflect synergistic alignment with the collective physicochemical signature of SRC-binding chemotypes, capturing emergent patterns that single-feature metrics cannot detect. Crucially, this assessment quantifies position within the SRC ligand manifold based on holistic molecular architecture, not binding affinity, or cellular potency. The KAL metric power derives from feature interdependence: aromatic ring count alone has limited discriminative value, but when contextualized by adjacent hydrogen-bond acceptors and specific topological constraints, it becomes a decisive element in the SRC ligand signature. This integrated approach enables rational navigation of chemical space toward SRC-like ligands, which is essential for interpretable generative design.

We deliberately adopted a binary (SRC vs. non-SRC) design rather than a multiclass kinome-wide classifier. This choice was motivated by two considerations: (i) the high structural homology of the ATP active site across kinase families renders fine-grained classification error-prone, and (ii) our generative design objective is a chemical scaffold transformation into the targeted SRC ligand design. KAL is implemented as a random forest classifier [110] trained on 20 RDKit-derived chemical descriptors [107,108] selected for their direct relevance to kinase ligand design. These chemical features can be classified into the following: (a) aromatic features: number of aromatic rings/carbocycles/heterocycles; (b) topological complexity: number of rings, aliphatic carbocycles/heterocycles, bridgehead atoms; (c) physicochemical properties: molecular weight, logP, QED, SAS, LabuteASA, Hall–Kier alpha; and (d) pharmacophoric elements: H-bond donors/acceptors, rotatable bonds, sp^3^ fraction, stereocenters, amide bonds. The training set comprises 1502 high-confidence SRC ligands (IC_50_ ≤ 100 nM) as positives and a balanced negative set of ~23,530 molecules, including ~9000 non-SRC kinase-associated ligands and ~14,530 drug-like background compounds (subsampled from GDB-17 to prevent class imbalance from overwhelming the minority SRC class). We opted to subsample GDB set to maintain model sensitivity to the minority class (SRC) since including all GDB molecules would create an extreme negative majority (~99% background), making the model trivially predict “0” and ignore the SRC class. The adopted split can also reflect a realistic chemical space where drug-like matter is abundant but not overwhelmingly dominant in screening libraries. Rather than attempting to distinguish among all kinase families—a task confounded by structural homology in the ATP active site—we focused exclusively on SRC kinase as a case study of generative chemical design.

The resulting score output represents the probability or “likelihood” that a molecule can be deemed an SRC kinase ligand. Values closer to 0 indicate that the molecule has a low KAL score whereas values closer to 1 indicate that the molecules have a high KAL score. To assess the performance of each model, Accuracy, Recall, Precision and F1 score were calculated to measure the performance of classification models. These parameters are defined as follows:

(2) $$Accuracy=\frac{TP+TN}{all};\;Precision=\frac{TP}{TP+FP}\;$$

(3) $$Recall=\frac{TP}{TP+FN};\;F_{1}=2\frac{Precision*Recall}{Precision+Recall}$$

F-score is a measure of precision and recall and is often used in binary classification problems. Precision is defined as the number of positive samples the model predicts correctly (true positives) divided by the true positives plus the false positives. Recall is defined as true positives divided by true positives plus false negatives. The model performance was evaluated using receiver operating characteristic area under the curve. The receiver operating curve (ROC) is a graph where sensitivity is plotted as a function of 1-specificity. The area under the ROC is denoted AUC. A reliable and valid AUC estimate can be interpreted as the probability that the classifier will assign a higher score to a randomly chosen positive example than to a randomly chosen negative example.

### 2.4. Bayesian Optimization for Global Exploration of Latent Space

We implemented a Bayesian optimization framework to navigate the 196-dimensional ChemVAE latent space in search of high-scoring SRC-like candidates, using the KAL score as the sole optimization objective. The protocol was configured as follows. The objective function is KAL score (range: 0–1), treated as a black-box, non-differentiable response function. No auxiliary constraints (e.g., QED, logP, SAS) were imposed, to isolate the effect of target-specific guidance. A surrogate model is employed in which Gaussian Process (GP) with a Matérn 5/2 kernel, is selected for its robustness to non-smooth response surfaces. Kernel hyperparameters were re-optimized via maximum marginal likelihood every 100 Bayesian optimization iterations. For acquisition function we used Expected Improvement (EI), which balances exploitation (refining high-KAL regions) and exploration (sampling high-uncertainty zones). The EI function was maximized using L-BFGS-B (Limited-memory Broyden–Fletcher–Goldfarb–Shanno algorithm), which is a quasi-Newton method for unconstrained or bound-constrained optimization. The EI acquisition function is smooth and differentiable (if the GP is smooth), and L-BFGS-B efficiently finds its local maximum in high-dimensional spaces, far faster than grid or random search.

To mitigate trapping in local optima, multiple optimization runs are launched from different random starting points sampled uniformly across the search space (e.g., the 196D ChemVAE latent box). Two independent Bayesian optimization simulations were executed. For unbiased Bayesian optimization 7000 latent vectors sampled uniformly from the full ChemVAE space and for biased Bayesian optimization 2258 latent vectors from high-confidence SRC ligands (IC_50_ ≤ 100 nM) + 4742 random vectors (total = 7000), to evaluate the impact of prior knowledge. For stopping criterion both types of simulations were terminated after 1500 acquisition steps, a threshold determined empirically from pilot runs to coincide with KAL score plateauing (ΔKAL < 0.001 over 100 consecutive steps). Each final latent vector was decoded 500 times using the ChemVAE decoder. A molecule was retained if at least one RDKit-sanitized SMILES was produced. No post-hoc re-scoring or property filtering was applied in order to preserve the integrity of the Bayesian optimization trajectory and enable unbiased analysis of generative limitations. This protocol ensures that observed effects reflect inherent constraints of the generative architecture, not artifacts of post-selection or multi-objective bias.

## 3. Results and Discussion

### 3.1. The Kinase Ligand Dataset and Its Embedding Reveals Organized Kinome Manifold in Latent Space

**Figure 1:**
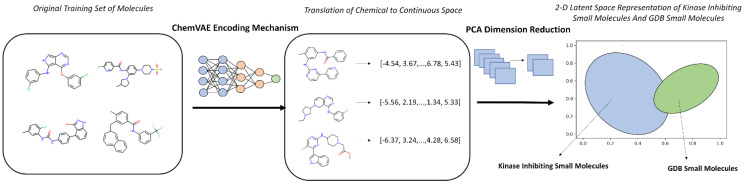
An Overview of Chemical to Continuous Space Translation using ChemVAE Encoding Mechanism.

This curated hybrid dataset comprising of ~220,000 diverse molecules forming a background set and ~60,000 available high-confidence PKIs from 37 distinct kinase families across the human kinome served as the training corpus for all machine learning components of our pipeline. Central to our approach was the ChemVAE architecture trained on SMILES strings that learns a continuous, low-dimensional latent representation of molecular structure. ChemVAE encodes each molecule into a fixed-length vector (here, 196-dimensional) by compressing its SMILES sequence through a bottleneck layer, while simultaneously optimizing for accurate reconstruction and property prediction (e.g., QED, logP, synthetic accessibility). This process effectively translates discrete chemical syntax into a differentiable geometric space, where semantic similarity (e.g., shared scaffolds or functional groups) is reflected in spatial proximity (Figure 1).

**Figure 2:**
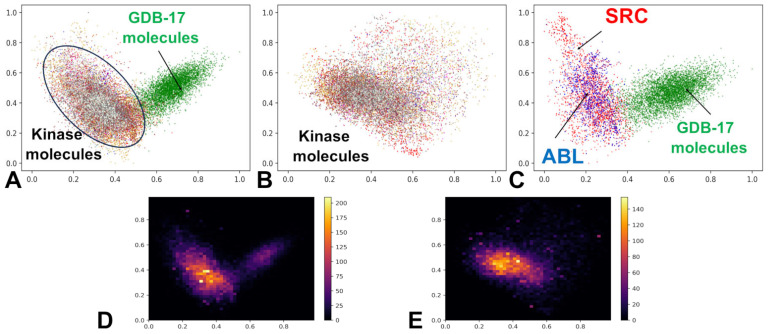
PCA and heatmaps of the latent spaces for GDB-17 small molecules and kinase ligands. (**A**) The 2-dimensional latent space representation of kinase molecules and GDB-17 small molecules dataset. Kinase molecules are shown in distinct colors for specific families, whereas GDB small molecules are shown in green dots. The locations of the latent space for these classes of molecules are pointed by arrows and annotated. (**B**) The 2-dimensional latent space representation of the kinase ligands from all 37 kinase families. The 10 major kinase families in the dataset are SRC (red), ABL1 (blue), EGFR (gold), CSF1R (orange), FLT3 (magenta), KDR (brown), LCK (turquoise), MAPK14 (gray), MET (honeydew). (**C**) The 2-dimensional latent space representation of the ABL kinase ligands (in blue) and SRC kinase-associated ligands (in red). (**D**) The 2-dimensional heatmap of latent space representation for GDB-17 molecules and kinase ligands from all studied kinase families. (**E**) The 2-dimensional heatmap of latent space representation for the kinase ligands. The density regions are color-coded with the high-density areas in yellow color, whereas low density regions tend towards purple.

To interrogate the organization of this latent space, we performed principal component analysis (PCA) on the encoded vectors and visualized the results in two dimensions (Figure 2). Embedding our large-scale kinase ligand dataset into the ChemVAE latent space revealed a striking and functionally meaningful organization: rather than scattering randomly, 60,000 kinase ligands spanning 37 families across the human kinome collapsed into a dense, low-volume manifold, sharply segregated from the diffuse cloud of 220,000 generic molecules (Figure 2A,B). The PCA projection revealed that despite their pharmacological diversity, kinase ligands collapsed into a dense, spatially contiguous cluster, sharply demarcated from the diffuse, cloud-like distribution of GDB molecules (Figure 2A).

This separation was not an artifact of labeling or sampling; it emerged naturally from the model’s unsupervised training on SMILES syntax, suggesting that molecular sequence intrinsically encodes functional semantics. This separation persisted even when examining kinase ligands in isolation, where sub-clustering by family was evident but incomplete, reflecting shared ATP-recognition motifs and overlapping chemotypes (Figure 2B). Within this kinase-rich region, a hierarchical structure became apparent. At the global level, all ATP-competitive ligands clustered together, reflecting the conserved architecture of the kinase catalytic cleft. Yet at a finer scale, family-specific subclusters emerged, highlighted for ABL and SRC kinase-associated ligands (Figure 2C).

The SRC family occupied the broadest region of latent space acting as a structural “hub” that overlapped significantly with LCK, ABL1, and EGFR. This proximity suggested that ABL, LCK and EGFR-derived molecules may be amenable to transformation into SRC-like chemotypes, a finding that would prove pivotal in our generative experiments. Visual inspection of the PCA-projected latent space revealed that most kinase ligands—regardless of target family—occupied a shared, high-density region that significantly overlapped with the clusters of SRC and ABL1 ligands (Figure 2B,C). This spatial co-localization suggests that, despite differences in selectivity and clinical indication, these molecules share a core set of chemical–functional features essential for recognition of the kinase ATP site, such as planar aromatic systems, hydrogen bond acceptors at the hinge region, and moderate molecular weight.

The emergence of highly skewed density peaks—with yellow indicating high concentration and purple low concentration—in the kernel density estimates (Figure 2D,E) demonstrated that kinase ligands occupy a statistically definable, low-volume manifold within the broader molecular landscape. High-density zones (Figure 2D,E) corresponded to chemically accessible regions, while sparse areas (purple) represented high-risk, low-validity territory. These high-density zones are not merely statistical artifacts; they represent chemically stable attractors in the latent space, where small local neighborhood samplings are more likely to decode into valid, synthesizable molecules. This topological organization provided the foundational rationale for a classification-based generative strategy: if kinase ligands form a separable region, a model trained to recognize that region could guide molecular generation toward it. This insight directly informed our subsequent generative strategies—both Bayesian optimization and cluster-guided local neighborhood sampling—which were explicitly designed to operate within or near these high-fidelity regions. To quantify this observation, we computed key statistical descriptors for each kinase family in the full 196-dimensional latent space, including the range (min–max), centroid (mean vector), and standard deviation across all dimensions (Table 1).

The results confirm that all kinase families span a remarkably similar domain in latent space, with minimum values ranging from −6.19 to −5.00 and maximum values from 5.97 to 7.06. This overlap reinforces the hypothesis that kinase ligands—by virtue of their shared target architecture—occupy a common, functionally constrained subspace within the broader chemical landscape.

Most notably, SRC ligands exhibited the largest spread in latent space, with the highest maximum standard deviation (1.632) and the broadest overall range (−5.89 to 6.20). This indicates that the SRC family encompasses the greatest structural diversity among the kinase classes studied, spanning a wider array of scaffolds, substitution patterns, and molecular topologies. In contrast, families like MAPK10 and MAPK14 showed more compact distributions (max SD: 1.295–1.298), suggesting greater structural homogeneity. This exceptional breadth has profound implications for generative design. The fact that SRC ligands dominate the latent region occupied by all kinase families implies that the chemical grammar of SRC association is representative of kinase recognition more broadly. Consequently, local neighborhood samplings applied to molecules from other kinase families—especially those with narrower distributions like FLT3 or MAPK10—may naturally evolve toward SRC-like chemotypes when steered toward high-density regions of the manifold. This positions SRC not just as a therapeutic target, but as a structural “hub” in kinase ligand space, making it an ideal focus for scaffold-hopping and family-to-family transformation strategies. These findings collectively demonstrate that the latent space not only captures functional similarity across kinase families but also encodes scaffold diversity in a quantifiable manner. The SRC family’s expansive footprint suggests it serves as a structural reservoir—a rich source of motifs that can be leveraged to transform ligands from other kinase classes into novel SRC-targeted candidates through guided latent space local neighborhood sampling. This finding motivated a dual-strategy generative campaign: one that explores the global manifold for novel, drug-like candidates (Bayesian optimization), and another that manipulates local neighborhoods to transform known scaffolds into new chemotypes (local neighborhood sampling-based engineering).

### 3.2. Multiclass and Binary Kinase Association Likelihood Classifiers

A core challenge in scaffold-aware generative design is the lack of an objective function that correlates chemical structure with target-specific functional plausibility without requiring costly physics-based scoring. The binary classification framework (SRC vs. non-SRC) achieved precision = 0.71, recall = 0.86, F1 = 0.78, with a macro F1-score of 0.88 (Table 2). The macro average precision score of 0.85 reinforces the overall satisfactory performance of the model because it means that the model was accurate in predicting if a given molecule was an SRC Kinase ligand 85% of the time. For classification models, an accuracy score of 0.85 is extremely strong. In addition, the macro average recall score of 0.92 validates the excellent performance of the model that the precision value helped establish. All these metrics indicate good classification performance of the model. This suggested that target-focused design may benefit from a simplified objective that avoids diluting signal across highly similar classes. In contrast, a parallel 10-class multiclass model performs markedly worse for SRC (F1 = 0.56; Table 3), confirming that simplification enhances signal in the presence of structural homology.

Critically, KAL is not intended to replace experimental validation. Its purpose is to provide a fast, interpretable, and chemically grounded guidance signal that enables rational navigation of latent space. As demonstrated in Section 3.3 and Section 3.4, KAL successfully steers both Bayesian optimization and local neighborhood sampling toward regions enriched in SRC-like molecules. However, its inability to recover multi-ring systems despite strong feature weighting exposes a key limitation—not of the scorer, but of the generative engine. This diagnostic clarity is precisely what a black-box model would obscure.

**Figure 3:**
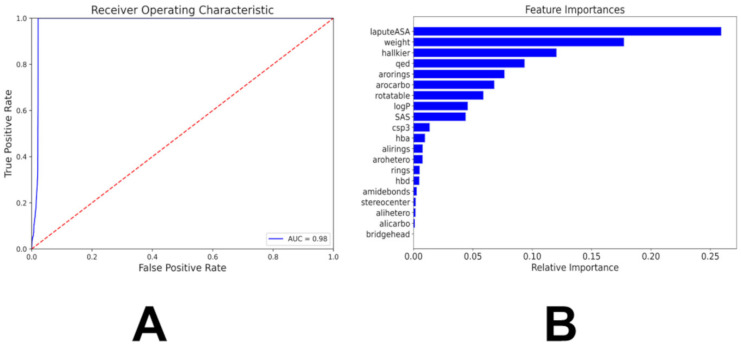
The performance and feature importance analysis of the chemical feature-based KAL classifier. (**A**) The Receiver Operating Curve (ROC) is a graph where sensitivity is plotted as a function of 1-specificity. The area under the ROC is denoted as AUC. The ROC–AUC graph measures the performance of the classifier in differentiating the kinase ligand molecules from GDB-17 small molecules (**B**) The feature importance analysis of the model. The importance of features is listed in descending order.

This reflects the inherent ambiguity in kinase ligand space as LCK and SRC ligands share overlapping scaffolds making fine-grained classification more error-prone. In the macro averages, the precision score was 0.63, the recall score was 0.59, and the F1-Score was 0.61. In the weighted average, the precision was 0.63, the recall score was 0.63, and the F1-Score was 0.63. The model showed the greatest metric values when predicting kinase ligands from the MAPK14 and MET kinase families. However, the other kinase families performed modestly in precision values, recall values or the F1-scores. In addition, the macro average F1-score of the multiclass model is 0.61 compared to the 0.88 F1-score of the binary model. Hence, the multiclass random forest model performs less favorably at distinguishing SRC ligands as compared to the chemical feature-based binary classifier. The chemical feature binary KAL classifier ca achieves the overall accuracy of distinguishing kinase inhibiting molecules around 98% (Figure 3). The AUC of the model was 0.98, indicating that the model can distinguish both classes with 98% certainty (Figure 3A). We performed feature importance analysis using scikit-learn mean decrease in Gini impurity metric (Figure 3B) which quantifies the contribution of each RDKit-derived descriptor to the classifier decision-making process. The top 10 features that contribute to the model prediction are the labute accessible surface area (labuteASA), weight, HallKier Alpha, the number of aromatic rings, aromaticity, the QED score, number of rotatable bonds, the logP score, the SAS score, and the number of hydrogen bond acceptors (Figure 3B).

KAL is used not only in classification, but also as a diagnostic and guiding signal for generative design implemented in the present investigation. In both Bayesian optimization and local neighborhood sampling-based latent space engineering approaches employed in our study, a reliable, differentiable (or at least efficiently evaluable) objective function is essential to direct search toward biologically relevant regions of chemical space. In the absence of such a function, generative models either produce random drug-like molecules or drift into chemically plausible but pharmacologically inert regions. For Bayesian optimization, KAL served as the black-box objective that the Gaussian process surrogate model sought to maximize. Bayesian optimization does not require gradients, but it does require a low-variance, high-signal scoring function that correlates with the desired property—in this case, SRC association potential. KAL fulfilled this role by providing a fast, interpretable, and chemically grounded estimate of targeted kinase recognition, enabling Bayesian optimization to iteratively select latent points predicted to yield high-KAL molecules without resorting to expensive physics-based scoring. For local neighborhood sampling-based generation, KAL played a diagnostic and filtering role. While local neighborhood samplings were guided by latent space geometry (cluster centroids), KAL was used to assess whether the transformed molecules had successfully migrated into the SRC ligands.

### 3.3. Bayesian Optimization Enables Efficient Exploration of SRC Kinase Ligand Chemical Space

**Figure 4:**
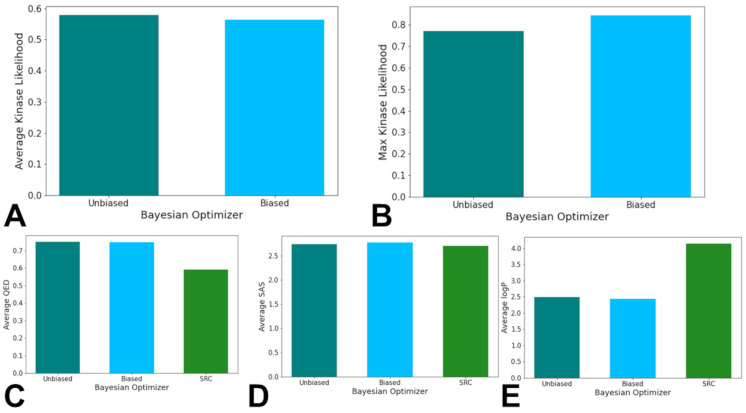
The average KAL scores of the molecules generated from the biased and unbiased Bayesian optimizer (**A**), the max KAL scores of the molecules generated from the biased and unbiased Bayesian optimizer (**B**), the average QED scores, (**C**), the average SAS scores (**D**) and the average logP scores (**E**) of the molecules generated from the biased and unbiased Bayesian optimizer, in comparison to the known SRC kinase-associated ligands. The unbiased histogram is in turquoise bars, the biased histogram is in light blue bars, and the SRC kinase ligand histogram is in green.

To systematically navigate the ChemVAE latent space in search of novel SRC kinase-associated ligands, we implemented a Bayesian optimization framework guided by the KAL scoring function. Bayesian optimization is a sequential design strategy that constructs a probabilistic surrogate model—here, a Gaussian process—to approximate an unknown objective function and iteratively selects new evaluation points by maximizing an acquisition function that balances exploration (sampling uncertain regions) and exploitation (refining high-scoring regions). In molecular design, this approach minimizes the number of costly function evaluations required to identify high-performing candidates. We executed two parallel optimization runs: unbiased Bayesian optimization, initialized with 7000 random latent points, and biased Bayesian optimization, first probed with 2258 known SRC ligands to inject prior knowledge of the target manifold before random initialization. Both performed 1500 acquisition steps. After decoding latent vectors to SMILES and filtering for validity using RDKit, biased Bayesian optimization yielded 492 valid molecules (83% validity), while the unbiased Bayesian optimization produced 390 (89% validity). Due to the random nature of the Bayesian optimizer, a threshold of KAL score of 0.5 was used as the baseline for a generated molecule to have a higher KAL score. Out of the valid molecules produced from each optimizer, 153 molecules out of the original 492 molecules produced, or 31.10%, from the biased optimizer had a calculated KAL value greater than 0.5. The unbiased optimizer maintained 145 of its original 390 valid molecules produced, or 37.18%, with a calculated KAL value greater than 0.5. When analyzing the molecules with a calculated KAL score greater than the 0.5 threshold, the unbiased optimizer had a higher average calculated KAL of 0.5783 compared to an average of 0.5639 for the molecules generated by the biased Bayesian optimizer (Figure 4A). The molecule with the highest calculated KAL score was produced by the biased Bayesian optimizer with a score of 0.8425. The molecule with the highest calculated KAL score produced by the unbiased optimizer had a score of 0.7693 (Figure 4B). Hence, the unbiased Bayesian optimization exhibited a higher average KAL among qualifiers (0.578 vs. 0.564), while the biased Bayesian optimization produced the single highest-scoring molecule (KAL = 0.8425) (Figure 4A,B). This duality suggested that unbiased exploration promoted consistent performance across chemical space, whereas bias enabled access to deeper local optima near known actives.

To evaluate the similarity testing metrics, we investigated the performance of each of the Bayesian optimizers based on average similarity scores of the generated molecules, as well as the maximum similarity score that each model produced. When analyzing all molecules generated from each Bayesian optimizer, the average maximum Tanimoto similarity scores computed against a reference set of 1502 high-confidence SRC kinase-associated ligands were 0.4656 and 0.4446 for the unbiased and biased Bayesian optimizers, respectively (Supporting Information, Figure S1A). The maximum Tanimoto similarity scores for the unbiased and biased Bayesian optimizers were 0.7115 and 0.7091, respectively (Supporting Information, Figure S1B). Strikingly, no generated molecule surpassed the conventional high-similarity threshold of 0.75. The maximum similarity was 0.7115 (unbiased) and 0.7091 (biased), and the top KAL molecule (0.8425) exhibited only modest similarity (0.548) (Supporting Information, Figure S1). When determining the performance of the Bayesian optimizers in relation to the chemical feature values of QED, logP, and SAS, the generated molecules from the optimizers had similar average SAS scores compared to the known SRC kinase-associated ligands but had significant differences in the average QED and logP scores. The average QED scores for the unbiased and biased Bayesian optimizers’ generated molecules were 0.7499 and 0.7486, respectively, in comparison to the known SRC kinase-associated ligands average QED score of 0.5908 (Figure 4C). The average logP scores for the unbiased and biased Bayesian optimizers’ generated molecules were 2.488 and 2.439, respectively, in comparison to the known SRC kinase-associated ligands average logP score of 4.137 (Figure 4D). The average SAS scores for the unbiased and biased Bayesian optimizers’ generated molecules were 2.742 and 2.772, respectively, in comparison to the known SRC kinase-associated ligands average SAS score of 2.706 (Figure 4E). The general similarity of the scores of the generated molecules in comparison to the known SRC kinase-associated ligands suggest that the metrics are being tuned as a part of the Bayesian optimizers’ hyperparameter tuning process. While there are differences between the generated molecules and the known SRC kinase-associated ligands when analyzing the QED and logP scores, the scores imply that the molecules produced by the Bayesian optimizers would be synthesizable and/or absorbable even with lower similarity metrics in other chemical features.

**Figure 5:**
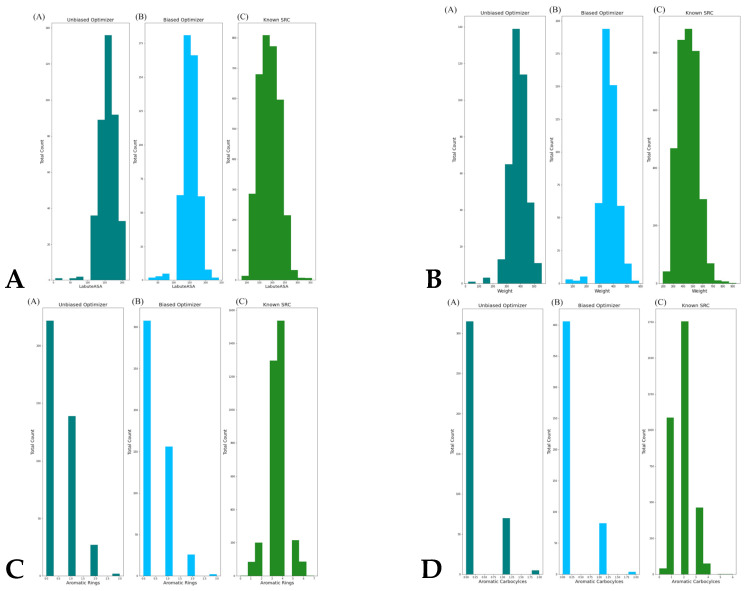
Histograms of the distribution of the LabuteASA values (**A**), the molecular weight (**B**), the number of aromatic rings (**C**) and the number of aromatic carbocycles (**D**) in the generated molecules using the unbiased Bayesian optimizer, the biased Bayesian optimizer, and compared to the set of known SRC kinase-associated ligands. The unbiased histogram is in turquoise bars, the biased histogram is in light blue bars, and the SRC kinase ligand histogram is in green.

Contrary to expectations, biasing the optimizer with known SRC ligands conferred no meaningful advantage in similarity, KAL, or structural plausibility. While the biased Bayesian optimization produced more valid molecules, its output exhibited markedly reduced scaffold diversity: the same two known SRC ligands repeatedly served as the nearest neighbors for the top generated molecules (Supporting Information, Figure S2). This pattern suggests that initial probing trapped the optimizer in a narrow local optimum, causing it to over-exploit motifs from only 1–2 reference compounds. In contrast, the unbiased Bayesian optimization generated structurally diverse candidates (Supporting Information, Figure S3), indicating broader exploration of chemical space. To dissect this discrepancy, we compared the distributions of the top KAL-informative features between generated molecules and real SRC ligands (Figure 5). LabuteASA and molecular weight were well-aligned: both optimizers produced molecules peaking at 150–200 Å^2^ and ~400 Da, closely mirroring the ~200 Å^2^ and ~500 Da peaks of real ligands (Figure 5A,B).

Most critically, aromatic complexity was severely underrepresented. Real SRC ligands show a broad distribution of 1–6 aromatic rings, with a strong peak at 3–4 rings—hallmarks of ATP-competitive binders that engage in π-stacking. In stark contrast, >80% of Bayesian optimization-generated molecules contained 0 or 1 aromatic ring, and none exceeded 3 rings (Figure 5C). A similar deficit was observed for aromatic carbocycles, where real ligands peak at 2 rings while generated molecules typically contain none (Figure 5D). Hence, Bayesian optimization excelled at tuning “drug-likeness” (QED, logP, SAS) but was not sufficiently robust at reproducing the topological grammar of kinase ligands. This suggests that Bayesian optimization constrained by SMILES-based latent space and the scalar KAL objective, could not effectively navigate to regions encoding multi-ring scaffolds.

In summary, Bayesian optimization successfully generated novel, valid, and drug-like molecules with moderate-to-high predicted SRC association potential. However, it systematically failed to recover the aromatic ring complexity that defines ATP-competitive kinase ligands—a failure that cannot be attributed to poor scoring, but to inherent limitations in the ChemVAE latent space. The results demonstrate that even a well-calibrated, interpretable scoring function like KAL cannot compensate for a generative engine that cannot access the relevant chemical subspaces.

### 3.4. Targeted Local Latent Neighborhood Sampling Recovers Pharmacophoric Complexity

While Bayesian optimization enabled efficient global sampling of the ligand manifold, it could not generate molecules with the multi-ring aromatic architectures characteristic of clinical SRC ligands. To address this, we further expanded on our earlier work [87] and developed a targeted latent space remodeling strategy that leverages the intrinsic organization of the ChemVAE embedding to guide scaffold transformation. This approach emphasizes guided exploration of high-density regions that are revealed in the latent space analysis contrasting random molecules with kinase ligands. Recognizing that kinase ligands form chemically coherent neighborhoods in latent space—even across distinct target families—we applied K-means clustering to partition the manifold into three structurally homogeneous regions, each enriched for shared scaffold motifs.

We used clustering in the latent space to find interpretable linear directions in the latent space that optimize the KAL score and enable morphing of kinase molecules into space of SRC kinase-associated ligands. In this approach it is assumed based on the latent space analysis that molecules with similar structures tend to cluster in the latent space and that interpolating two molecules x1 and x2, represented by latent vectors z1 and z2, can lead to intermediate molecules whose structures gradually change from x1 to x2. Since molecular structures correlate with molecular properties, these assumptions imply that molecules with comparable properties would cluster together and interpolating two molecules with different values of the molecular property could lead to gradual changes in molecular structures. By performing cluster-based analysis in the latent representation of the molecules, the generative design approach encourages ChemVAE to explore the high-density distinct areas of the latent space for molecule generation while also facilitating morphing of the kinase molecules from different families into SRC kinase-associated ligands. In this approach, the properties of generated molecules can be controlled by sampling latent representations along linear directions to optimize the KAL metric.

**Figure 6:**
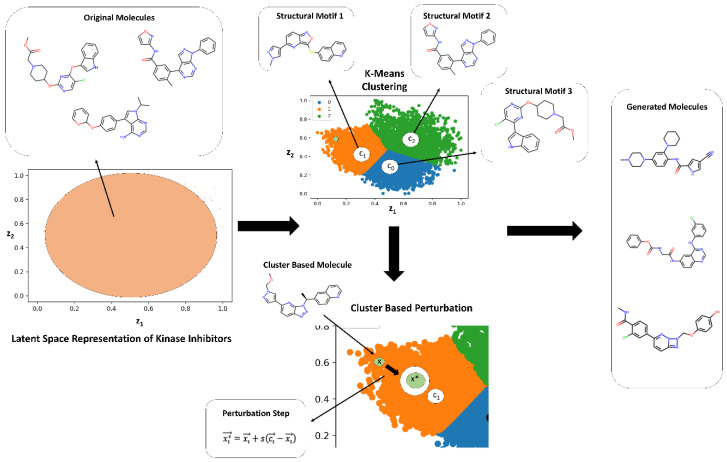
A schematic workflow of the cluster-based targeted remodeling design implementation. K-Means clustering is applied in the latent space, where different clusters represent specific molecular characteristics. The 3-cluster split is represented by the graph on the right, where the colors of blue, green, and orange indicate the 3 clusters, respectively. The centroids of each cluster, depicted by the labels of c_0_, c_1_, and c_2_, function as the representative of the structural motifs and molecular properties of that cluster. Utilizing the centroid, we modify our input by employing local neighborhood sampling, as shown in the local sampling step, where c represents the centroid, x represents the original encoded molecule, and x* represents the molecule after local neighborhood sampling step. This implementation alters the encoded input such that it converges towards the centroid, and in turn, generates molecules close to the specific motifs of the respective cluster. After the input is modified with the local sampling step, ChemVAE decodes the latent space areas and produces a set of new molecules.

The targeted latent space remodeling strategy includes non-biased and biased changes to the latent space. First, molecules in a non-biased manner are clustered into groups allowing molecules with comparable properties to gather. We assume that the molecules clustered for each cluster contain certain molecular and chemical properties. To then transform these molecules, we invoke a controllable step of cluster-based local neighborhood sampling. Using the centroid of each cluster as the representative of the properties, we navigate every data point in the cluster closer to the centroid by optimizing a set of parameters. By implementing a cluster-based local neighborhood sampling, we efficiently explore and navigate the latent space along interpretable and controllable directions yielding a diverse set of novel molecules and causing various molecular scaffolds to emerge. It is worth noting that the resulting score/output of the feature-based KAL classifier represents the probability that a molecule can be deemed as an SRC kinase ligand. The produced molecules are evaluated with the classifier during targeted latent space remodeling and when the probability output > 0.7 we refer to these molecules as potential SRC kinase-like ligands as according to the classifier the generated molecules would have >70% chance to belong this category (Figure 6).

During the cluster-based stage of the process, 1500 encoded molecules from different kinase families were selected and processed through a series of experiments to obtain the optimal parameters of the targeted remodeling scheme that leads to a high yield of valid generated molecules, while simultaneously achieving the objective of transforming the kinase molecules to potential SRC kinase-associated ligands. The three main parameters of the clustering in the latent space were evaluated and optimized to ensure optimal generation of valid molecules: the number of clusters assigned, the value of the scaling factor in the local neighborhood sampling, and the optimal level of noise. We found that a 3-cluster based split, with a scaling factor s=0.8  for the centroid-based remodeling, and a noise level of 5.0 provided the optimal set of parameters to guarantee a high generation yield of valid and novel compounds. Within each cluster, we performed targeted local sampling where molecules were shifted incrementally toward the cluster centroid using a controlled interpolation (scaling factor *s* = 0.8) and minimal stochastic noise (Figure 6). This directed navigation preserved chemical validity while steering generation toward high-density zones rich in pharmacophoric features. The approach yielded a three-fold increase in valid output compared to random sampling and often recovered multi-ring aromatic systems that were systematically absent in Bayesian optimization outputs.

**Figure 7:**
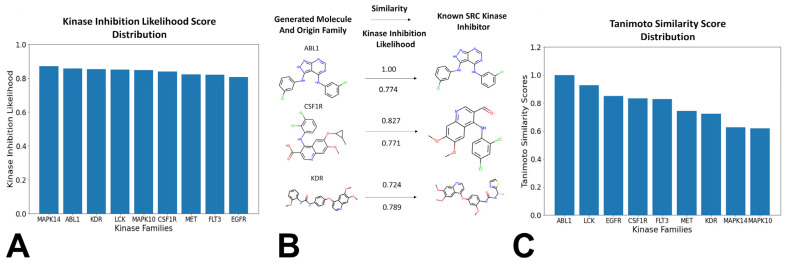
The analysis of the generated molecule output with respect to KAL score and Tanimoto similarity. (**A**) The KAL distributions of the generated molecules originated from ligands from every kinase family. The horizontal axis displays the kinase families from which the generated molecules originate from. The vertical axis displays the KAL score ranging from 0 to 1, where a score of 1 indicates the high KAL and a score close to 0 indicates the lowest KAL. (**B**) A visual representation of the generated molecules along with the respective molecular metrics. On the left, the generated molecules, and their originating family that they were transformed from are shown. On the right, the corresponding known SRC kinase ligand with the high similarity to the generated molecule. (**C**) The distribution of similarity scores with respect to the known SRC kinase-associated ligands for the generated molecules originated from ligands of different families. The horizontal axis represents the originating families from which these molecules were transformed. The vertical axis represents the similarity score from 0 to 1, where a score of 1 indicates perfect similarity to the comparison molecule and 0 corresponds to high degree of dissimilarity.

We also investigated the distribution of the generated molecules featuring the high KAL scores (>0.75) as a function of the originated kinase family (Figure 7A). Strikingly, it was observed that the perturbation-based approach can produce novel valid molecules with the high KAL probability when the generative process originates from known ligands targeting any of the explored kinase families. This indicates that a combination of clustering and perturbation-based targeted exploration of the latent space allows for efficient chemical transformation of existing kinase molecules from all represented families. To evaluate similarity between the generated molecules and known SRC kinase-associated ligands, we examined the fraction of the generated molecules with the high Tanimoto similarity coefficient values. The Tanimoto similarity coefficient is a metric that compares the molecular similarity of two compounds using Morgan fingerprint analysis [111]. Molecules with Tanimoto coefficient values that are above 0.75 are considered to have high similarity with the reference molecule.

Interestingly, the generated molecules originated from LCK ligands produced the largest fraction of novel kinase-like compounds (~40%) with the high similarity to the SRC kinase-associated ligands. We also observed that the generated molecules initiated from ligands of ABL1, LCK and EGFR produced the dominant number of kinase-like novel molecules with the highest similarity coefficients to known SRC ligands (Figure 7B). It is worth noting that the generated molecules originated from ligands of ABL1 and LCK yielded the highest similarity scores with SRC ligands, with most molecules displaying Tanimoto similarity coefficient > 0.8. The SRC/ABL and SRC/LCK duality of many kinase drugs is well recognized, most notably exemplified by dual SRC/ABL drugs Dasatinib and Ponatinib.

**Figure 8:**
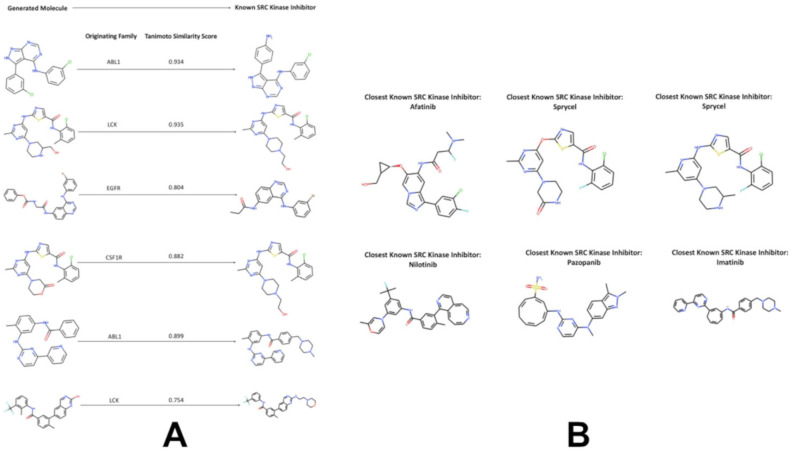
A sample of generated molecules with (**A**) high Tanimoto similarity score to the known SRC kinase-associated ligands and closest to the FDA approved SRC kinase drugs (**B**).

In addition, we found that the generated molecules originated from ligands of EGFR, CSF1R, FLT3, and MET families also produced good similarity to the known SRC ligands. These findings may imply that local neighborhood navigation of the latent space that optimized directionality of exploration based on the KAL score could facilitate generation of valid molecules in different areas of the latent space. Indeed, a substantial number of the generated molecules emerged from mapping connections in the latent space between SRC, LCK and ABL ligands. At the same time, the algorithm facilitated efficient sampling of the latent space and corresponding transformations of the kinase ligands targeting other families into molecules with both the high KAL score and the high similarity to the SRC ligands. This process also enabled cross-family scaffold transformation: LCK and EGFR ligands, which occupy regions of latent space proximal to SRC, showed the highest conversion efficiency (19–23% of total output), whereas MAPK14 and FLT3 contributed minimally (3–7%). LCK and MAPK10 emerged as the most productive sources of unique, high-similarity candidates, suggesting that certain kinase scaffolds possess inherent “plasticity” for repurposing into SRC-targeted leads. Our results revealed the important role of the LCK family, which accounts for ~40% of all high-similarity outputs, far surpassing other families. This is not a sampling artifact but reflects a genuine topological affinity between LCK and SRC inhibitor spaces, directly enabled by our guided remodeling approach. To illustrate the output of the generative pipeline, we compiled a list of several representative generated SRC-like kinase molecules that originated from the ligands of different kinase families. These molecules were characterized by the high KAL and a considerable similarity to the existing SRC kinase-associated ligands (Figure 8A).

We noticed that some of the novel valid molecules with the highest similarity to the SRC ligands were produced starting from the latent space regions of the ABL1 and LCK kinase ligands. A sample of generated molecules reflected both the diversity of molecular scaffolds and high degree of synthetic feasibility that were enabled through local remodeling approach (Figure 8). Molecules originating from the EGFR and LCK clusters—families known for quinazoline and pyrrolopyrimidine scaffolds—were successfully remodeled into novel chemotypes containing 3–5 aromatic rings, including quinazoline- and pyrimidine-like cores characteristic of clinical SRC ligands (Figure 8B). Importantly, these remodeled molecules maintained physiologically relevant logP values (2–4) indicating better preservation of the drug-like features (Supporting Information, Figures S4–S8). This correlates with pharmacophoric retention: 87% of high-KAL local sampling candidates preserved ≥3 critical kinase recognition motifs (e.g., adenine-mimetic rings, H-bond acceptors at C7), versus only 32% for Bayesian optimization-generated molecules.

These findings reveal a fundamental distinction in generative design paradigms. Bayesian optimization follows a “property-first” paradigm: it optimizes global chemical properties under the assumption that molecular plausibility correlates with target relevance. This approach succeeds for flexible targets but fails for kinases, where function is dictated by precise topological constraints. In contrast, guided local sampling adopts a “scaffold-first” philosophy: by anchoring generation in structurally coherent neighborhoods, it preserves critical topological features even as novel chemotypes emerge. Nevertheless, both methods are constrained by the underlying representation: ChemVAE learns a continuous manifold, but its SMILES-based decoding often fails to preserve complex ring topologies during interpolation. The latent space contains the seeds of molecular complexity, but the generative engine frequently collapses these features during reconstruction.

Collectively, our results demonstrate that scaffold-aware molecular generation requires three essential elements: (1) neighborhood-aware generation to preserve topological integrity, (2) multi-parameter optimization balancing chemical properties, and (3) representation systems that inherently respect structural constraints. Critically, no amount of scoring refinement can overcome a generative engine that cannot access critical chemical subspaces—a constraint we term the “representation gap.”

## 4. Discussion

This study presents a modular, interpretable, and chemistry-first framework for the de novo design of SRC kinase-associated ligands, integrating deep generative modeling (ChemVAE), a chemically grounded scoring function (KAL), probabilistic optimization (Bayesian optimization), and scaffold-aware latent space local neighborhood sampling. Across two complementary strategies, global exploration via Bayesian search and local transformation via cluster-guided engineering—we generated novel, drug-like molecules with moderate-to-high predicted SRC association potential. A central finding is that kinase ligands occupy a distinct, low-volume manifold in the latent space, segregated from general drug-like matter. Within this region, SRC ligands exhibit the highest structural diversity, acting as a “hub” that overlaps with other kinase families. This organization was learned implicitly from SMILES syntax, suggesting that the latent space effectively encodes functional semantics. Notably, LCK-derived molecules showed the highest propensity for transformation into SRC-like candidates. This validates the use of latent space geometry as a map for rational scaffold hopping, identifying SRC as a privileged target for cross-family repurposing. Our comparative analysis further clarifies methodological trade-offs. Biased Bayesian optimization (seeded with known SRC actives) converged prematurely to narrow structural clusters, while unbiased optimization yielded more diverse, higher-scoring candidates. On the other hand, cluster-guided local sampling best preserved pharmacophoric features but remained constrained by SMILES decoding limitations.

We utilized a SMILES-based VAE as a transparent diagnostic platform. By using a modular framework, we could independently probe the influence of representation, scoring, and search strategy. Both Bayesian optimization and local neighborhood sampling-based generation systematically failed to recover the multi-ring aromatic systems characteristic of many ATP-competitive kinase ligands. Most generated molecules contained three or fewer rings, even though aromaticity was a heavily weighted feature in the KAL scoring function. This suggests a fundamental limitation in the generative engine: SMILES-based VAEs often struggle to decode complex ring topologies, regardless of the optimization pressure applied. This finding underscores that even a perfect scoring function cannot compensate for a generative model that cannot access the relevant chemical subspace. Our results, including the systematic underrepresentation of multiple aromatic rings or the privileged transformability of LCK into SRC-like chemotypes, reveal relevant structural and functional truths about kinase ligand space that would be masked in end-to-end pipelines. By exposing representational gaps and showcasing scaffold-aware navigation of latent space, this study argues for hybrid systems that combine the diagnostic transparency of interpretable AI frameworks with the generative power of modern architectures.

## 5. Conclusions

This study establishes a diagnostic framework for evaluating the capabilities and limitations of scaffold-aware generative models in representing structurally complex SRC-binding scaffolds. By analyzing the organization of kinase-targeted chemical space in latent representations, we reveal three critical insights for computational molecular design. First, latent space geometry encodes functional relationships between kinase families. SRC-binding scaffolds form a structural “hub” within the latent manifold, with LCK-derived molecules demonstrating 2-4× higher transformability to SRC-like chemotypes than other families. This organization provides a predictive map for rational scaffold transformation, enabling targeted redesign of molecular scaffolds across kinase families. Second, we expose a fundamental representation gap that cannot be overcome by scoring refinement alone. Despite ring count being a top feature in our structural similarity metric, SMILES-based generative models systematically fail to access multi-ring topologies characteristic of kinase-binding scaffolds. This limitation stems not from optimization strategy but from the inherent constraints of sequential molecular representations, where complex ring topologies are entangled across latent dimensions. Third, our comparative analysis demonstrates that hybrid generative strategies are essential for scaffold-aware design. Unbiased exploration yields greater diversity and higher average structural similarity scores, while cluster-guided local sampling preserves critical topological features. Biasing optimization with known actives traps search in narrow local optima without improving structural quality—a finding that challenges conventional wisdom in generative chemistry. By positioning this work as a diagnostic analysis of representation capabilities rather than a biophysical validation study, we provide a methodological benchmark for current AI frameworks and a blueprint for next-generation hybrid systems. The future of computational molecular design lies not in algorithmic novelty alone, but in topology-aware representations that preserve structural complexity while integrating chemical principles. Our framework establishes that diagnosing representational limits—rather than merely generating novel molecules—is the critical foundation for advancing scaffold-aware molecular design in the post-deep learning era.

## Figures and Tables

**Table 1:** **Statistical Distributions of Kinase Families in the 196-Dimensional Latent Space** *.

Family	Min Range	Max Range	Min Average	Max Average	Min Stand Dev	Max Stand Dev
ABL1	−5.89215	5.97272	−1.34594	1.2609	0.78482	1.46389
**SRC**	**−5.89215**	**6.20087**	**−1.38016**	**1.30248**	**0.86567**	**1.63218**
CSF1R	−5.19233	6.84467	−1.19730	1.21217	0.65711	1.46416
EGFR	−6.18875	6.55361	−1.25954	1.22010	0.82409	1.39603
FLT3	−5.00162	6.45221	−1.17921	1.15374	0.69147	1.42987
KDR	−6.15671	7.05822	−1.37088	1.32073	0.80067	1.35351
LCK	−6.15671	6.62534	−1.38279	1.39623	0.81684	1.55863
MAPK10	−5.08671	5.98541	−1.16237	1.14753	0.68575	1.29511
MAPK14	−6.15671	6.89392	−1.52617	1.44791	0.73652	1.29781
MET	−6.13674	6.49813	−1.45546	1.52347	0.79279	1.53428

* Reported values are aggregated across all latent dimensions. Standard deviation (Stand Dev) reflects the spread of each family’s embedding distribution.

**Table 2:** Binary Chemical Feature-Based Classification.

	Precision	Recall	F1-Score	Support
0	0.99	0.98	0.98	23,530
1	0.71	0.86	0.78	1502
Macro Avg	0.85	0.92	0.88	25,032
Weighted Avg	0.97	0.97	0.97	25,032

**Table 3:** Multiclass Classification Chemical Feature-Based Classification.

	Precision	Recall	F1-Score	Support
ABL1	0.51	0.58	0.55	409
**SRC**	**0.57**	**0.56**	**0.56**	**660**
CSF1R	0.69	0.54	0.61	142
EGFR	0.69	0.74	0.71	795
FLT3	0.55	0.46	0.50	194
KDR	0.58	0.59	0.58	916
LCK	0.47	0.41	0.44	313
MAPK10	0.77	0.55	0.64	163
MAPK14	0.75	0.80	0.78	722
MET	0.74	0.72	0.73	421
Macro Avg	0.63	0.59	0.61	4735
Weighted Avg	0.63	0.63	0.63	4735

## Data Availability

Data are fully contained within the article and Supplementary Materials and are available in the Github website. All scripts, software and models used in the experiments are available in the GitHub site https://github.com/kassabry/Kinome-Scale-Generative-Modeling (accessed on 1 January 2026) that provides detailed documentation and guides for the deposited information and software.

## Supplementary Materials

The following supporting information can be downloaded at: https://www.mdpi.com/article/10.3390/biom16020209/s1. Supplementary Materials include Supplementary Figures S1–S8. Supplementary Materials also contain the following additional information: the original set of generated molecules produced using Bayesian optimization, and cluster-guided local neighborhood sampling approaches along with the calculated physical–chemical features. Figure S1. The average similarity scores to the known SRC kinase inhibitors (A) and maximum similarity to the known SRC kinase inhibitors (B) of all generated molecules from the Unbiased (in turquoise bars) and Biased Bayesian Optimizers (in light blue bars). Figure S2. The Top Three Molecules Generated from the Biased Bayesian Optimizer with the Closest Known SRC Kinase Inhibitors. Figure S3. The Top Three Molecules Generated from the Unbiased Bayesian Optimizer with the Closest Known SRC Kinase Inhibitors. Figure S4. The Distribution of Average Aromatic Rings for Generated Molecules from the top 10 Originating Kinase Families. Figure S5. The Distribution of Average Number of Hydrogen Bond Acceptors for Generated Molecules from the top 10 Originating Kinase Families. Figure S6. The Distribution of Average Number of Hydrogen Bond Donors for Generated Molecules from the top 10 Originating Kinase Families. Figure S7. The Distribution of Average Number of Average Molecular Weight for Generated Molecules from the top 10 Originating Kinase Families. Figure S8. The Distribution of Average Number of Average Number of Rotatable Bonds for Generated Molecules from the top 10 Originating Kinase Families.
