# Supplementary material for: From Latent Manifolds to Targeted Molecular Probes: An Interpretable, Kinome-Scale Generative Machine Learning Framework for Family-Based Kinase Ligand Design
Source: Biomolecules. 2026 Jan 29;16(2):209. doi: 10.3390/biom16020209 (PMC12938821; doi:10.3390/biom16020209)
Supplement: Supplementary file 1 [file biomolecules-16-00209-s001.zip › biomolecules-4015436-supplementary.pdf]

From Latent Manifolds to Functional Probes: An Interpretable, Kinome-Scale Generative Machine Learning Framework for Family-Targeted Kinase Inhibitor Design

Ryan Kassab[1,2] , Keerthi Krishnan,[1] Gennady Verkhivker[1,3,4]

[1]Keck Center for Science and Engineering, Graduate Program in Computational and Data Sciences, Schmid College of Science and Technology, Chapman University, Orange, CA 92866, United States of America

[2]Department of Biostatistics and Medical Informatics , School of Medicine and Public Health, University of Wisconsin-Madison, Madison, WI 53726 United States of America

[3]Department of Biomedical and Pharmaceutical Sciences, Chapman University School of Pharmacy, Irvine, CA 92618, United States of America

[4]Department of Pharmacology, Skaggs School of Pharmacy and Pharmaceutical Sciences, University of California San Diego, 9500 Gilman Drive, La Jolla, CA 92093, United States of America

* Correspondence: verkhivk@chapman.edu; Tel.: +1-714-516-4586 (G.V)

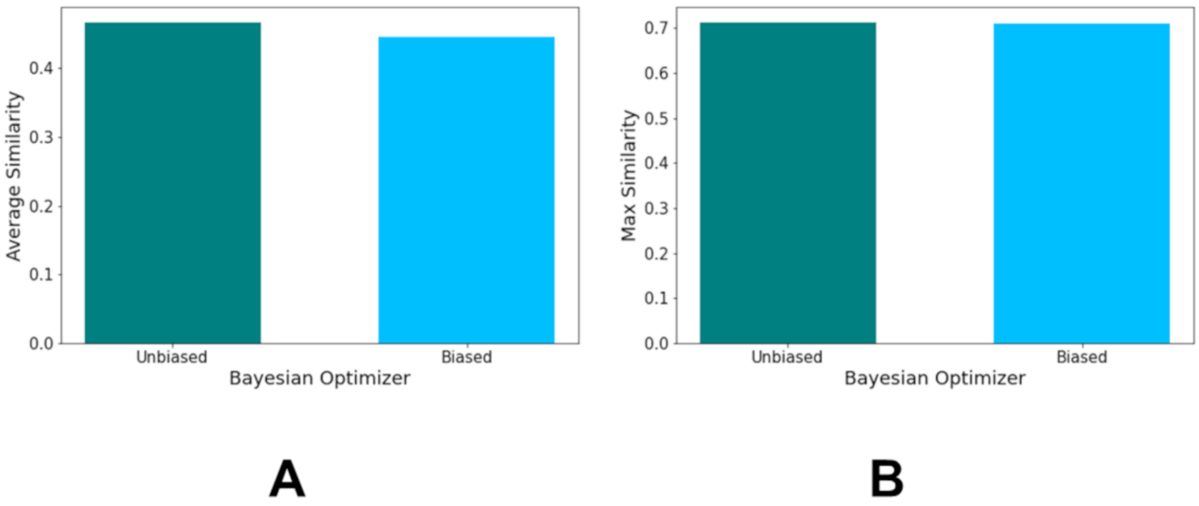

Figure S1. The average similarity scores to the known SRC kinase inhibitors (A) and maximum similarity to the known SRC kinase inhibitors (B) of all generated molecules from the Unbiased (in turquoise bars) and Biased Bayesian Optimizers (in light blue bars)

Generated Molecule	Known SRC Kinase Inhibitor	Tanimoto Similarity Score	Kinase Inhibition Likelihood	QED Score	logP Score	SAS Score
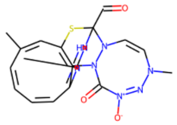	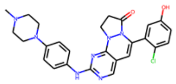	0.70910	0.57519	0.63193	1.87408	3.15589
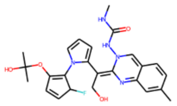	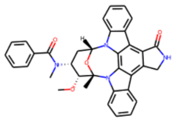	0.64016	0.75332	0.62254	4.24315	2.2926
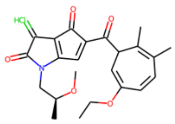	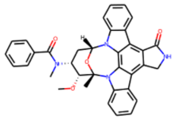	0.63440	0.58261	0.78757	0.51124	2.80652

Figure S2. The Top Three Molecules Generated from the Biased Bayesian Optimizer with the Closest Known SRC Kinase Inhibitors

Generated Molecule	Known SRC Kinase Inhibitor	Tanimoto Similarity Score	Kinase Inhibition Likelihood	QED Score	logP Score	SAS Score
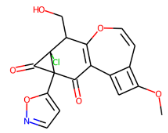	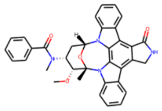	0.71153	0.58567	0.63763	3.6400	2.3322
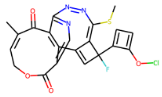	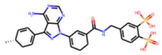	0.69567	0.59816	0.71623	4.24062	1.80823
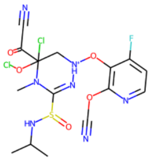	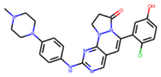	0.66666	0.60690	0.76593	-0.15078	3.17604

Figure S3. The Top Three Molecules Generated from the Unbiased Bayesian Optimizer with the Closest Known SRC Kinase Inhibitors

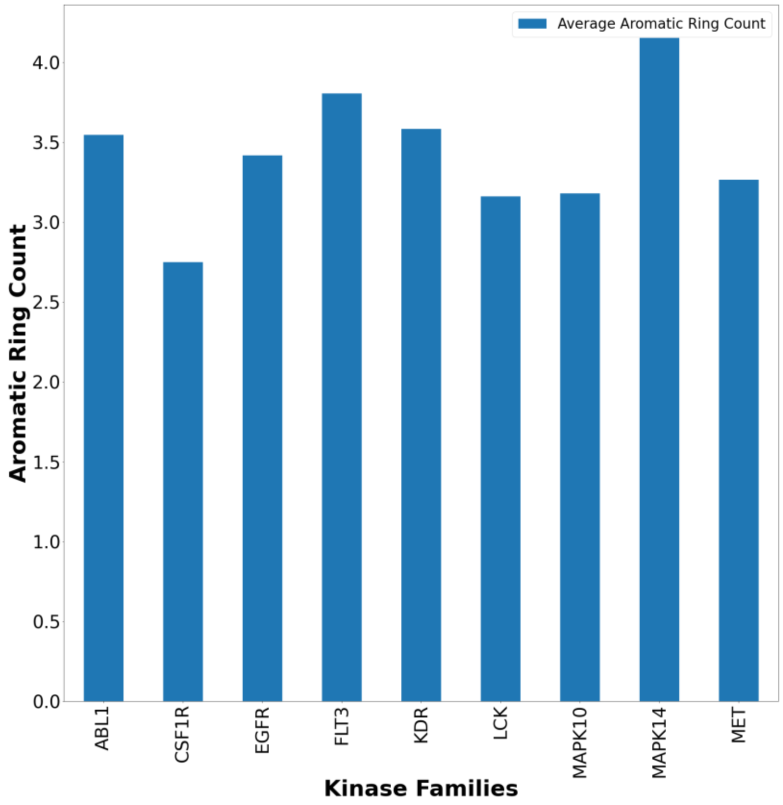

Figure S4. The Distribution of Average Aromatic Rings for Generated Molecules from the top 10 Originating Kinase Families.

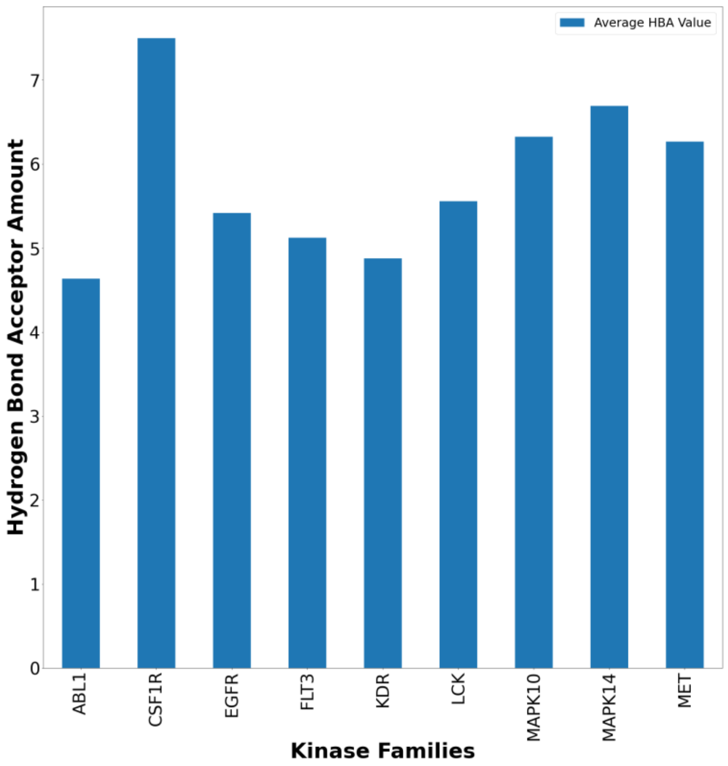

Figure S5. The Distribution of Average Number of Hydrogen Bond Acceptors for Generated Molecules from the top 10 Originating Kinase Families.

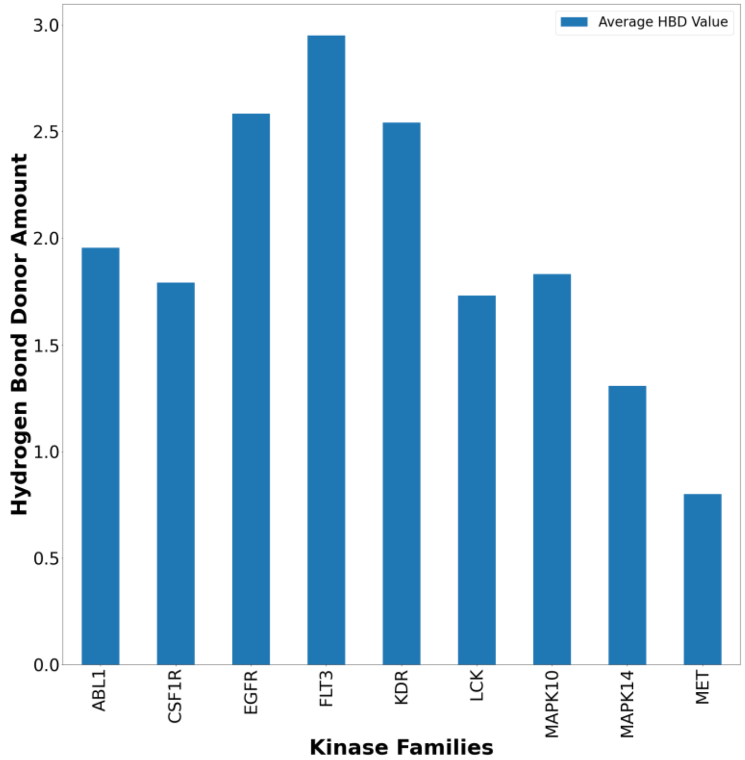

Figure S6. The Distribution of Average Number of Hydrogen Bond Donors for Generated Molecules from the top 10 Originating Kinase Families.

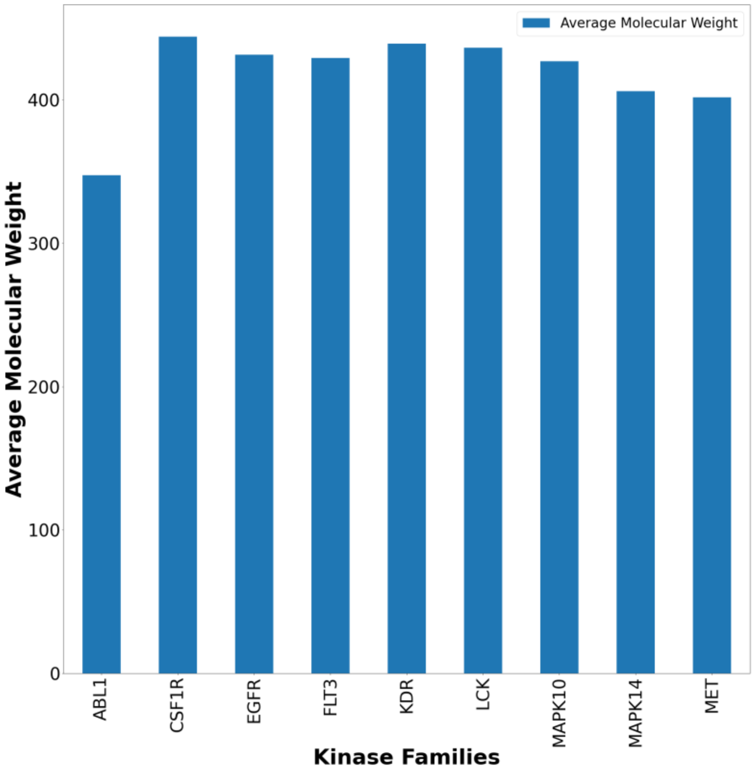

Figure S7. The Distribution of Average Number of Average Molecular Weight for Generated Molecules from the top 10 Originating Kinase Families.

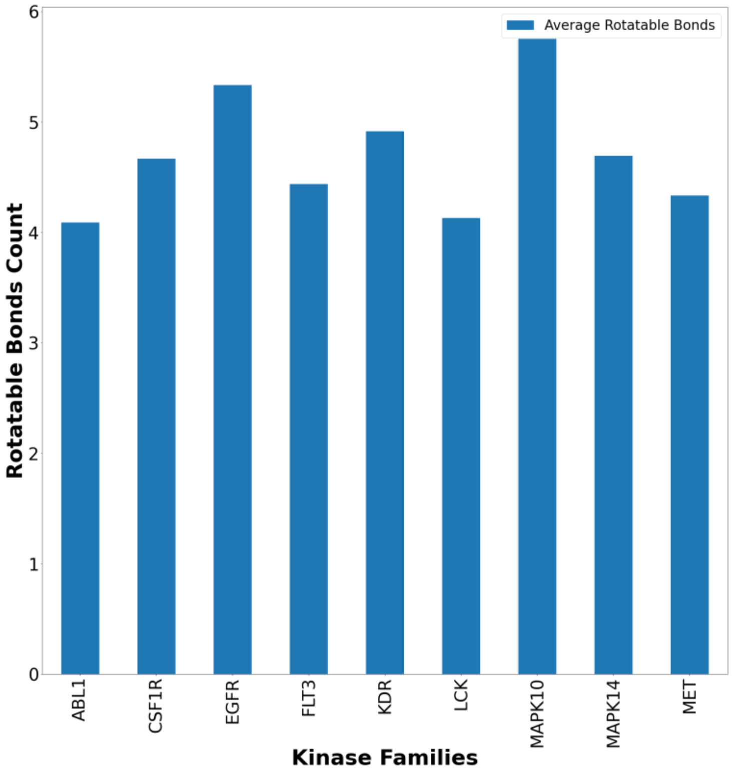

Figure S8. The Distribution of Average Number of Average Number of Rotatable Bonds for Generated Molecules from the top 10 Originating Kinase Families.
